# The effects of urbanization on global *Plasmodium vivax* malaria transmission

**DOI:** 10.1186/1475-2875-11-403

**Published:** 2012-12-05

**Authors:** Qiuyin Qi, Carlos A Guerra, Catherine L Moyes, Iqbal R F Elyazar, Peter W Gething, Simon I Hay, Andrew J Tatem

**Affiliations:** 1 Department of Geography and Emerging Pathogens Institute, University of Florida, Gainesville, FL, USA; 2 Spatial Ecology and Epidemiology Group, Tinbergen Building, Department of Zoology, University of Oxford, South Parks Road, Oxford, UK; 3 Eijkman-Oxford Clinical Research Unit, Jalan Diponegoro No. 69, Jakarta, 10430, Indonesia; 4 Department of Geography and Environment, University of Southampton, Highfield, Southampton, UK; 5 Fogarty International Center, National Institutes of Health, Bethesda, MD, 20892, USA

**Keywords:** *Plasmodium vivax*, Urbanization, Dominant *Anopheles* vectors, Mapping

## Abstract

**Background:**

Many recent studies have examined the impact of urbanization on *Plasmodium falciparum* malaria endemicity and found a general trend of reduced transmission in urban areas. However, none has examined the effect of urbanization on *Plasmodium vivax* malaria, which is the most widely distributed malaria species and can also cause severe clinical syndromes in humans. In this study, a set of 10,003 community-based *P*. *vivax* parasite rate (*Pv*PR) surveys are used to explore the relationships between *Pv*PR in urban and rural settings.

**Methods:**

The *Pv*PR surveys were overlaid onto a map of global urban extents to derive an urban/rural assignment. The differences in *Pv*PR values between urban and rural areas were then examined. Groups of *Pv*PR surveys inside individual city extents (urban) and surrounding areas (rural) were identified to examine the local variations in *Pv*PR values. Finally, the relationships of *Pv*PR between urban and rural areas within the ranges of 41 dominant *Anopheles* vectors were examined.

**Results:**

Significantly higher *Pv*PR values in rural areas were found globally. The relationship was consistent at continental scales when focusing on Africa and Asia only, but in the Americas, significantly lower values of *Pv*PR in rural areas were found, though the numbers of surveys were small. Moreover, except for the countries in the Americas, the same trends were found at national scales in African and Asian countries, with significantly lower values of *Pv*PR in urban areas. However, the patterns at city scales among 20 specific cities where sufficient data were available were less clear, with seven cities having significantly lower *Pv*PR values in urban areas and two cities showing significantly lower *Pv*PR in rural areas. The urban–rural *Pv*PR differences within the ranges of the dominant *Anopheles* vectors were generally, in agreement with the regional patterns found.

**Conclusions:**

Except for the Americas, the patterns of significantly lower *P*. *vivax* transmission in urban areas have been found globally, regionally, nationally and by dominant vector species here, following trends observed previously for *P*. *falciparum*. To further understand these patterns, more epidemiological, entomological and parasitological analyses of the disease at smaller spatial scales are needed.

## Background

The world population has undergone unprecedented growth along with rapid urbanization. Slightly more than 50% of the population (3.6 billion) is now living in urban areas compared to only 30% (0.7 billion) in 1950 [1]. By 2050, it is projected that urban dwellers will account for approximately 67% (6.3 billion) of the world total population, while most of the estimated growth will be concentrated in less developed regions, particularly in Asia and Africa [1]. These substantial transitions have significant public health implications associated with changes in the social and physical environment and access to public health services [2-6].

Although large heterogeneity exists, it is commonly accepted that the process of urbanization reduces malaria transmission, primarily because urban environments (e.g. the lack of suitable breeding sites, the pollution of existing larval habitats, etc.) are generally unsuitable for malaria vectors [7-9]. Other explanations include better access to health care services and an increased ratio of humans to mosquitoes [7,10,11]. However, there is concern regarding urban malaria in less developed regions, typically those undergoing rapid and unprecedented urbanization [12,13].

Between the two dominant parasite species of human malaria, *Plasmodium falciparum* has attracted the focus of most research because of its high mortality and intensive transmission in Africa [14]. *Plasmodium vivax* malaria, in contrast, is commonly considered as a “benign” infection and largely overlooked by researchers, government, and funding agencies. Increasing evidence has shown that *P*. *vivax* is neither rare nor benign, however. It is estimated that 2.85 billion people were at risk of *P*. *vivax* infection in 2009, with 91% (2.59 billion) of them living in Central and South East Asia [15], and that *P*. *vivax* is the most widely distributed (geographically) malaria species of humans. Furthermore, although the infection with *P*. *vivax* malaria is rarely directly fatal, it can cause severe clinical syndromes [16,17].

Recent studies have examined the impact of urbanization on *P*. *falciparum* malaria endemicity and disease burden estimation [7-9,13,18]. Various urban extent maps have been used to compare the differences in *P*. *falciparum* malaria endemicity between urban settlements and rural areas [18]; exclude the urban extents of cities identified as malaria free in the mapping of malaria transmission limits [19,20]; downgrade endemic classes in estimates of malaria burden [9,21]; and predict *P*. *falciparum* malaria endemicity based on geo-statistical models [22,23]. However, according to our best knowledge, no known research has examined the effect of urbanization on *P*. *vivax* malaria over similarly large scales. In addition, the regions of highest *P*. *vivax* transmission in Asia are composed of a considerably greater range of vector species and species complexes than seen in Africa, where *P*. *falciparum* transmission is princi-pally concentrated [24-27], and urbanization may impact each of these vector species differently, dependent on their preferences and bionomics. For example, *Anopheles culicifacies* was reported to be the vector responsible for 60-65% malaria cases in urban environments of India [28] and shows significant environment tolerance and adaptability [29,30], while larvae of *Anopheles stephensi* were found in various domestic containers and collections of water related to construction and industrial sites in cities [31,32]. Therefore, there is a need to examine the effects of urbanization on *P*. *vivax* transmission by dominant vector species to discern whether differential impacts are evident.

Here geo-referenced *P*. *vivax* parasite rate (*Pv*PR) surveys and urban extent maps are integrated to examine the impact of urbanization on *P*. *vivax* malaria transmission at various spatial scales (global, regional, national and at the city level). Furthermore, distribution maps of dominant *Anopheles* vectors are used to explore the relationships between urbanization, *Anopheles* vectors and *P*. *vivax* malaria transmission.

## Methods

### Datasets

#### The MAP *Pv*PR database

**Figure 1:**
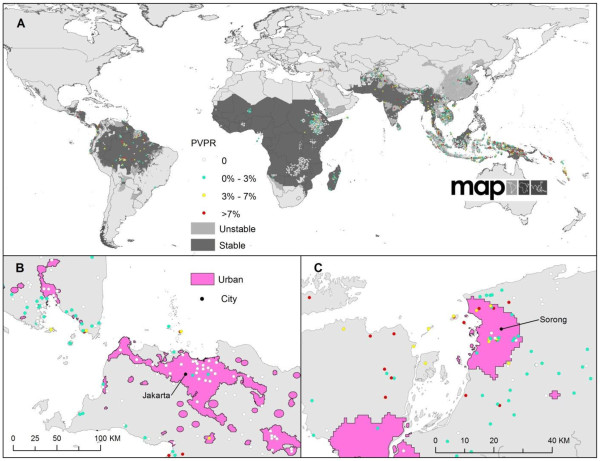
**The global spatial limits of *P *.*vivax *malaria transmission in 2009[**[15]**].** Panel **A** shows the spatial limits of *P*. *vivax* malaria risk defined by *P*. *vivax* annual parasite incidence (*Pv*API) data. Areas were defined as stable (dark grey, where *Pv*API ≥0.1 per 1,000 pa), unstable (medium grey, where *Pv*API < 0.1 per 1,000 pa) and no risk (light grey, where *Pv*API = 0 per 1,000 pa). The community-based *Pv*PR surveys are plotted and colored based on their values (red, where *Pv*PR >7%; yellow, 3% < *Pv*PR <7%; light blue, *Pv*PR < 3%) with zero-valued surveys shown in white. Panel **B** and Panel **C** are close-ups for regions with plenty of *Pv*PR surveys with Panel B showing the area around Jakarta, Indonesia and Panel **C** showing the areas around Sorong, Indonesia.

As with *P*. *falciparum* malaria, parasite rate (PR) is the most commonly reported and consistent metric of *P*. *vivax* malaria endemicity [33]. A total of 10,003 community-based *P*. *vivax* parasite rate (*Pv*PR) surveys taken between 1985 and 2010 were obtained by the Malaria Atlas Project (MAP [34]). The logistically intensive process of searching for, identifying and geo-locating the *Pv*PR surveys has been documented elsewhere [35]. All these *Pv*PR surveys were geo-referenced to precise locations and not duplicated within three months at the same site. A summary of some of the key features of the *Pv*PR survey data is presented in Table 1. Of the surveys, 410 (4.1%) were in the Americas, 1,651 (16.5%) in Africa, Saudi Arabia and Yemen (Africa+), and 7,942 (79.4%) in Central and South East Asia (Asia+). Approximately half (51%) of the *Pv*PR values are zero and the majority of the surveys were undertaken after 2000. The sample sizes of these surveys varies, with most of them (76%) are being larger than 50. Among the 95 *P*. *vivax* malaria endemic countries (*Pv*MECs) [15], *Pv*PR data were available for 53 (12 in the Americas, 19 in Africa+ and 22 in Asia+). There are 8,588 discrete *Pv*PR survey locations and the distribution of them are shown in Figure 1, overlaid on the international limits of *P*. *vivax* malaria transmission [15], with most of the survey points located in Southeast Asia and the Horn of Africa.

**Table 1:** **Summary of the *Pv*PR surveys by region**

	**Africa+**	**Americas**	**Asia+**	**Total**
***Pv*PR values**				
Number of zero records	1,299	193	3,631	5,123
Mean *Pv*PR (%)	0.60	3.25	3.55	3.05
Median *Pv*PR (%)	0.00	0.61	0.51	0.00
**Time period of surveys**				
1985-1999	225	223	1,328	1,776
2000-2010	1,426	187	6,614	8,227
**Sample size**				
1-50	911	151	1,316	2,378
>50	740	259	6,626	7,625
Median (IQR)	48 (34–109)	87 (37–210)	120 (67–281)	107 (53–236)
**Records of surveys**				
GRUMP-UE defined urban	444	61	755	1,260
GRUMP-UE defined rural	1,203	349	7,241	8,743
Discrete geographic locations	1,424	291	6,873	8,588
Total	1,651	410	7,942	10,003

Africa+ =Africa, Saudi Arabia and Yemen; Asia+ =Central and South East Asia.

#### Global urban map

Although urbanization has been one of the most important transformations of our world for decades, there is still little consensus on the definitions of what consists an urban area and urbanization among national and international bodies [2]. Such ambiguity has lead to the construction of several global urban maps (e.g., Digital Chart of the World (DCW) [36], Global Rural Urban Mapping Project (GRUMP) [37], Advanced Very High Resolution Radiometer (AVHRR) Global Land Cover Classification urban land cover class [38], Defense Meteorological Satellite Program-Operational Linescan System (DMSP-OLS) [39], and Moderate Resolution Imaging Spectroradiometer (MODIS) Land Cover Product Binary Data [40,41]) derived from satellite imagery [42]. As discussed in Tatem et al. [42], all of these global urban maps demonstrated a different range of inaccuracies and limitations, however, the GRUMP urban extent map was considered to be most accurate in matching original urban assignments of *P*. *falciparum* malaria surveys [18]. Therefore, the GRUMP urban extent (GRUMP-UE) map was used here to distinguish *Pv*PR surveys taken in urban areas from those in rural areas. This urban extent map was developed by the Centre for International Earth Science Information Network (CIESIN) at 1 km × 1 km spatial resolution in 2004, utilizing information from satellite night-time lights (NTL), Landsat satellite sensor imagery and other geographical data [43].

#### Dominant *Anopheles* vector maps

The distributions and bionomics of dominant *Anopheles* vectors play an important role in malaria transmission and are the targets of vector control [44]. Vector species normally display a range of ecological and behavioural characteristics. For example, unlike other malaria vectors, an urban environment is favored by the “urban vector” *Anopheles stephensi*[45]. To assess the impact of urbanization on *P*. *vivax* malaria transmission by dominant *Anopheles* vectors, expert-opinion distribution maps of global dominant vector species (DVS) of malaria were obtained from the Malaria Atlas Project [25-27,34]. These maps were constructed through exhaustive searches of literature and refinement through opinion and experience by *Anopheles* experts [27]. A total of 41 maps of DVS were available, of which, 19 were in the Asia-Pacific region [26], 13 in Africa, Europe and the Middle East [25], and nine in the Americas [27].

### Analysis

#### Urbanization and *P*. *vivax* malaria transmission

To quantify the patterns of *P*. *vivax* malaria transmission between urban and rural areas at global, regional and national scales, sets of spatially and temporally associated urban–rural pairs of *Pv*PR values were obtained and tested. Firstly, all the geo-referenced *Pv*PR surveys were overlaid onto the GRUMP-UE map to derive an urban/rural assignment. Following previous approaches [18], for each *Pv*PR survey assigned as urban, all the rural *Pv*PR surveys taken within 100 km and five years were identified. Then, the identified rural *Pv*PR values were averaged and assigned to that urban *Pv*PR survey to make spatially and temporally associated urban–rural *Pv*PR value pairs [18]. Given the highly skewed distribution of *Pv*PR values in the MAP database [35], the Wilcoxon Signed Rank [46], a nonparametric test for paired variables, was used to determine if significant differences between *Pv*PR values in urban and rural areas existed. These tests were undertaken globally, by region (Africa+, Americas, Asia+) and by country (those for which at least ten urban–rural *Pv*PR survey pairs existed) to examine if the patterns of *P*. *vivax* malaria transmission between urban and rural areas were significant.

As the choice of spatial and temporal limits (100 km and five years) is arbitrary in obtaining urban–rural pairs of *Pv*PR values, a robustness analysis was conducted. Sets of urban–rural *Pv*PR pairs were obtained through applying various spatial and temporal limits (100 km and two years; 50 km and five years; 50 km and two years), and tested under the Wilcoxon Signed Rank test, respectively. In addition, the mean number of rural surveys paired to each urban survey and the overlap rate (∑ number of rural surveys paired to each urban survey/total number of rural surveys) for each spatial and temporal limit were calculated to assess the effects of overlapping rural surveys in the sample pairs.

To examine local variations (city scale) in *Pv*PR, groups of *Pv*PR surveys inside individual city extents (urban) and surrounding areas (rural) were identified and tested. Cities where more than eight *Pv*PR surveys (to provide a reasonable number of cities for testing) fell inside their urban extents were first identified. For each city, rural *Pv*PR surveys that fell within 100 km of the centroid of the urban extent were found and assigned to that city. Following this, for each city, *Pv*PR values within its urban extent and surrounding rural area were compared and tested using the Wilcoxon Rank Sum test.

#### Dominant *Anopheles* vectors

The impact of urbanization on malaria endemicity may vary by dominant *Anopheles* vectors of human malaria. To test this, *Pv*PR values between urban and rural areas within the extents of 41 dominant *Anopheles* vector were examined.

Sets of spatially and temporally associated urban–rural pairs of *Pv*PR values within the extents of each dominant *Anopheles* vector were extracted and tested separately. The geo-referenced *Pv*PR surveys were firstly overlaid onto the GRUMP-UE map to derive an urban/rural assignment. For each dominant *Anopheles* vector, all the *Pv*PR surveys that fell within its extent were extracted. For each urban *Pv*PR survey, all of the rural *Pv*PR surveys taken within 100 km and five years were again identified, averaged and assigned to that urban *Pv*PR survey to make a set of spatially and temporally associated urban–rural *Pv*PR value pairs [18]. This set of urban–rural *Pv*PR value pairs were then subject to the Wilcoxon Signed Rank test to determine if significant differences in *Pv*PR between urban and rural areas existed.

## Results

### Urbanization and *P*. *vivax* malaria transmission

Among the *Pv*PR surveys, 1,260 were classified as urban and 8,743 were classified as rural based on the GRUMP-UE map (Table 1). The mean sample size was 278 for the urban surveys and 230 for the rural surveys, which are comparable. Table 2 shows the results of the Wilcoxon Signed Rank tests between urban and rural pairs of *Pv*PR values defined by GRUMP-UE. Significantly higher *Pv*PR values in rural areas were found globally and in the Africa+ and Asia+ regions, while in the Americas, significantly lower values of *Pv*PR in rural areas were found. The *Z* values indicate, however, that the differences observed in the Americas are weaker than in other regions. Moreover, the numbers of surveys available were much smaller in the Americas.

**Table 2:** **Results of Wilcoxon Signed Rank tests on *Pv*PR values between GRUMP-UE defined urban (U) and rural (R) survey pairs for countries, regions and the World**

**Region**	**No. pairs**	**U > R**		**U < R**		**Z**	**P-value**
		**No. pairs**	**Rank sum**	**No. pairs**	**Rank sum**		
**Africa+**	**428**	**33**	**1,432**	**86**	**5,708**	**−5.670**	<0.001***
Ethiopia	80	18	587	61	2,573	−4.853	<0.001***
Sudan	192	7	47	9	89	−1.086	0.286
Yemen	35	7	41	13	169	−2.389	0.018**
Other countries	13	1	2	3	8	−1.095	0.361
**Americas**	**49**	**23**	**636**	**19**	**263**	**2.307**	0.021**
Brazil	22	15	135	3	36	2.156	0.032**
Mexico	10	1	10	9	45	−1.784	0.067*
Other countries	17	7	72	7	33	1.224	0.232
**Asia+**	**712**	**127**	**50,971**	**517**	**156,719**	**−11.194**	<0.001***
Afghanistan	68	23	1,023	42	1,122	−0.323	0.749
Bangladesh	27	1	27	26	351	−3.892	<0.001***
China	26	8	146	18	205	−0.749	0.461
Indonesia	462	74	18,926	328	62,077	−9.256	<0.001***
India	26	4	60	22	291	−2.933	0.003***
Cambodia	12	1	6	11	72	−2.589	0.007***
Nepal	18	2	35	16	136	−2.199	0.021**
Pakistan	11	6	38	5	28	0.444	0.700
Thailand	12	1	2	11	76	−2.903	0.001***
Vietnam	23	0	0	21	231	−4.014	<0.001***
Other countries	29	7	124	17	176	−0.743	0.466
**World**	**1,189**	**183**	**84,784**	**622**	**239,631**	**−11.732**	<0.001***

Africa+ =Africa, Saudi Arabia and Yemen; Asia+ =Central and South East Asia (*** = P < 0.01, ** = P < 0.05, * = P < 0.1).

Those countries with at least ten urban–rural *Pv*PR value pairs and the other countries combined for each region (Africa+, Americas and Asia+) were tested further and the results are presented in Table 2. The trends found in most of the countries in Africa+ (Ethiopia, Yemen) and Asia+ (Bangladesh, Indonesia, India, Cambodia, Nepal, Thailand, Vietnam) were consistent with the global and regional findings, with significantly lower values of *Pv*PR in urban areas. The relationships found between urban and rural *Pv*PR values for the other countries in Africa+ (Sudan and other African countries) and Asia+ (Afghanistan, China, Pakistan and other Asian countries) were not significant. There are two countries (Ghana and Zambia) in Africa that have sufficient *Pv*PR surveys but are of entirely zero values, so were not listed. The results for the Americas are certainly not as conclusive as the relationships found in the other regions, with one country (Brazil) showing significant higher urban *Pv*PR values, another country (Mexico) showing the reverse and the other American countries showing insignificant differences, though each were only based on a small number of *Pv*PR pairs.

The robustness analysis (Table 3) suggests that the overlap rate of rural surveys decreases as the spatial and temporal limits contract, while the patterns of *Pv*PR between urban and rural areas at global and regional scales are generally consistent. Thus, the method used to determine the relationship of *P*. *vivax* malaria transmission between urban and rural areas is robust and the effects of overlapping rural surveys on the results are minimal.

**Table 3:** **Robustness analysis of the Wilcoxon Signed Rank tests on urban–rural *Pv*PR value pairs derived from various spatial and temporal limits**

**Region**	**100 km 5 years**		**100 km 2 years**		**50 km 5 years**		**50 km 2 years**	
	**Z**	**P-value**	**Z**	**P-value**	**Z**	**P-value**	**Z**	**P-value**
Africa+	−5.670	<0.001***	−5.623	<0.001***	−5.644	<0.001***	−5.397	<0.001***
Americas	2.307	0.021**	1.680	0.094*	0.486	0.631	0.730	0.471
Asia+	−11.194	<0.001***	−11.065	<0.001***	−9.080	<0.001***	−9.005	<0.001***
World	−11.732	<0.001***	−11.555	<0.001***	−10.052	<0.001***	−9.757	<0.001***
No. pairs	1,189		1,106		1,156		1,106	
Mean No. R	49.653		42.287		31.813		27.061	
Overlap rate	6.752		5.349		4.206		3.423	

Africa+ =Africa, Saudi Arabia and Yemen; Asia+ =Central and South East Asia; Mean No. R = Mean number of rural surveys for each urban–rural pair; Overlap rate = Σ number of rural surveys paired to each urban survey/total number of rural surveys (*** = P < 0.01, ** = P < 0.05, * = P < 0.1).

**Figure 2:**
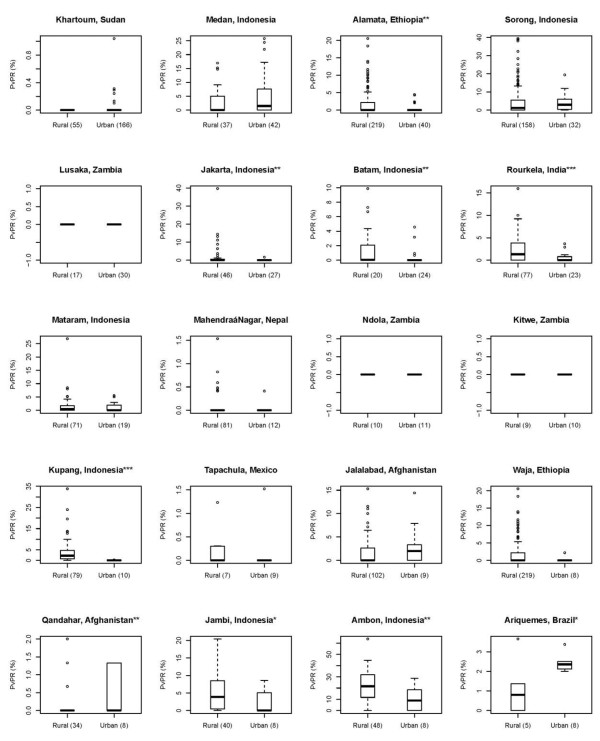
**Boxplots showing the differences in *Pv *PR values between GRUMP-UE defined urban and rural surveys for cities.** (*) denotes the significant level of the test results (*** = P < 0.01, ** = P < 0.05, * = P < 0.1).

Figure 2 shows the boxplots for urban and rural *Pv*PR surveys for individual cities whose extents were defined by the GRUMP-UE. The results indicate that the patterns among the 20 cities examined were less consistent with the global, regional and national patterns found. Seven cities (Alamata, Ethiopia; Jakarta, Batam, Kupang, Jambi and Ambon, Indonesia; Rourkela, India) were found to have significantly lower *Pv*PR values in their urban extents than the surrounding rural areas; two cities (Qandahar, Afghanistan; Ariquemes, Brazil) were found to have significantly lower *Pv*PR values in their surrounding rural areas (though again, the numbers of surveys were small). The remainder were either insignificant or of zero *Pv*PR values.

### Dominant *Anopheles* vectors

**Figure 3:**
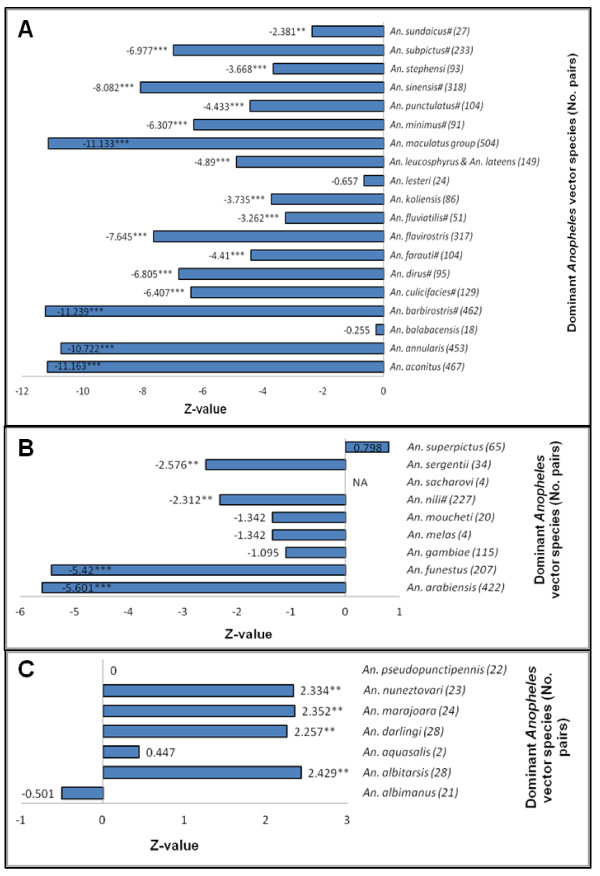
**Bar charts showing the test results for the dominant *Anopheles *vectors of human malaria.** Panel **A** shows the results of Wilcoxon Signed Rank tests on *Pv*PR values between GRUMP-UE defined urban (U) and rural(R) survey pairs for the dominant *Anopheles* vectors of human malaria in Asia-Pacific region. Panel **B** shows the results for the dominant *Anopheles* vectors in Africa, Europe and the Middle East. Panel **C** shows the results of Wilcoxon Signed Rank tests for the dominant *Anopheles* vectors in the Americas. (^#^) denotes that a vector species is now recognized as a species complex. (*) denotes the significant level of the test (*** = P < 0.01, ** = P < 0.05, * = P < 0.1).

Figure 3A presents the results of Wilcoxon Signed Rank tests on *Pv*PR values between GRUMP-UE defined urban and rural survey pairs stratified by the dominant *Anopheles* vectors of human malaria in the Asia-Pacific region. In this region, the patterns of lower *P*. *vivax* malaria transmission in urban areas are noticeable and consistent, with significantly higher rural *Pv*PR values found for most of the dominant *Anopheles* vector distributions (17 out of 19). Furthermore, insignificant differences between urban and rural areas (*Anopheles balabacensis* and *Anopheles lesteri*) were found in regions with small numbers of survey pairs.

Figure 3B shows the results of Wilcoxon Signed Rank tests on *Pv*PR values between urban and rural survey pairs stratified by the dominant *Anopheles* vector distributions in Africa, Europe and the Middle East. *Pv*PR surveys were only available for nine (of the 13) dominant *Anopheles* vectors. The consistent patterns of lower *Pv*PR values in urban areas are not as evident as in Asia-Pacific region. The differences of *Pv*PR between urban and rural areas are found to be statistically significant for only four (out of nine) dominant *Anopheles* vectors (*Anopheles arabiensis*, *Anopheles funestus*, *Anopheles nili* and *Anopheles sergentii*). The others were insignificant, while two of them (*Anopheles melas* and *Anopheles sacharovi*) have insufficient number of *Pv*PR surveys.

Figure 3C presents the results of Wilcoxon Signed Rank tests for analyses stratified by dominant *Anopheles* vectors in the Americas. For two (out of nine) of the dominant *Anopheles* vectors no *Pv*PR surveys fell within their extents. Unlike the patterns exhibited in the other regions, consistently higher *Pv*PR values in urban areas were observed in this region, with most of the dominant *Anopheles* vectors (*Anopheles albitarsis*, *Anopheles darlingi*, *Anopheles marajoara* and *Anopheles nuneztovari*) showing significantly higher urban *Pv*PR values. However, the numbers of survey pairs in this region are generally small.

More detailed statistical results for the three regions are provided in Additional file 1.

## Discussion

The rapid urban transformation of the developing world [47] has and will continue to have a profound influence on the malaria landscape. The need for accurate and contemporary descriptions of populations at risk (PAR) has lead to several attempts to quantify the impact of urbanization on *P*. *falciparum* malaria transmission [9,13,18]. Knowledge is lacking however regarding the relationship between urbanization and *P*. *vivax* malaria transmission. In this study, the most contemporary and comprehensive database of *Pv*PR surveys was used to explore the differences in *P*. *vivax* transmission between urban and rural areas.

Lower *P*. *vivax* malaria transmission in urban areas than surrounding rural areas was found globally, and in the Africa+ and Asia+ regions (Table 2), which corroborates previous findings that the urban environment is typically not suitable for malaria mosquito vectors [7-9]. The consistent patterns of significantly lower urban *Pv*PR values found at the national scale in most of the countries in Africa+ and Asia+ further supports these findings (Table 2). However, the urban–rural survey pairs for each region are dominated by a few countries (e.g., Indonesia accounts for 65% of the Asia pairs and Sudan accounts for 45% of the Africa pairs), which make the patterns found at regional scale less informative. Distinct and inconsistent results were found in the Americas, with higher *Pv*PR values in urban areas at the continental scale and for one particular country (Brazil) at the national scale. This result is probably due to the lack of *Pv*PR surveys in this region, as surveys from the region only account for 4.1% of the *Pv*PR global database. Getting extreme results is more likely when the numbers of surveys are small and only the rural *Pv*PR surveys were averaged. There is also evidence suggesting that higher malaria transmission in some areas of Brazil was actually a result of rapid urbanization, during which settlements were built close to forest boundaries or along riversides and thus resulting in greater exposure to the malaria parasite for residents [48].

Figure 2 indicates that considerable heterogeneity exists when examining individual cities, with two cities (out of twenty) showing significantly lower *Pv*PR in their surrounding rural areas, and seven cities showing significantly lower prevalence in urban areas. Thus, only nine of the twenty cities examined showed significant differences in transmission between urban and rural areas, and three showed zero prevalence both within and around the urban areas. Compared to *P*. *falciparum*[18], therefore, the patterns of *Pv*PR between urban and rural areas exhibit a higher level of heterogeneity. Several possible reasons include: 1) the wider transmission limits of *P*. *vivax*[15], but lower transmission intensity with many zero *Pv*PR values in the database; 2) the wide distribution in Asia and high prevalence of Duffy negativity in Africa [49,50]; 3) relatively fewer *Pv*PR surveys available in the MAP database compared with a total of 22,212 *P*. *falciparum* parasite rate (*Pf*PR) surveys in 2010 [23].

The *Pv*PR differences between urban and rural settings within the ranges of the dominant *Anopheles* vectors generally follows the patterns found in each region. This is partly because vector species that had sufficient urban–rural *Pv*PR pairs within their extents usually cover a large portion of the region. An issue raised here is that the distributions of most of the vector species overlap substantially with each other. Thus, drawing conclusions about the patterns of individual vector species is difficult without considering such overlap. However, according to expert-opinion distribution maps of global DVS [25-27], the spatial relationships among those vector species are extremely complex and the interaction effects of them are beyond the scope of this analysis.

The GRUMP-UE was used to define urban areas here, though several alternative global urban maps exist [42]. Every global map suffers from different errors and uncertainties [42], and the GRUMP-UE map exhibits overestimation of large urban area extents, due to the blooming effect of NTL imagery [42,51]. This suggests that the *Pv*PR urban values that were significantly higher than nearby rural ones found in the Americas and several other individual cities could actually be located in surrounding lower population density areas, as significantly higher malaria prevalence and entomologic inoculation rates in peri-urban areas compared to urban centers have been found in a number of studies [9,13,18]. To assess briefly this potential bias in the GRUMP-UE map, urban extents mapped using Moderate Resolution Imaging Spectroradiometer (MODIS) satellite sensor imagery [40,41] were utilized to derive an alternative, more conservative, urban assignment for the *Pv*PR surveys. Again, sets of spatially and temporally associated urban–rural pairs of *Pv*PR values were extracted and tested. The results show that, due to the more conservative nature of the classification, and the fact that only intensely urban areas were mapped [40,41], far fewer *Pv*PR surveys were identified as urban and the differences in *Pv*PR between urban and rural areas were generally not significant (see Additional file 2). Such results highlight the differing outcomes that can occur through using differing definitions of urban, and that the effects of urbanization on *P*. *vivax* transmission may extend beyond the borders of intensely urban areas for most of the regions as a general trend of decreased *Pv*PR was found in urban areas. Another issue is that the GRUMP-UE map was produced in 2004 and some *Pv*PR surveys may be misclassified as the urban extent changes through time. However, global urban maps that are updated regularly or that quantify urban extent change do not currently exist. Furthermore, the majority of the *Pv*PR surveys were conducted between 2000 and 2010 (Table 1). Thus, it is reasonable to use the single time-point GRUMP-UE map in this analysis.

A range of human-induced environmental changes (e.g., deforestation, urbanization, water control projects and climate change) have been identified as drivers of ‘emerging’ and ‘reemerging’ diseases and the transmission of vector-borne and other infectious diseases [52-55]. Urbanization is usually recognized as one of the primary factors affecting vector-borne diseases [56] as it can not only provide residents with better access to healthcare and interventions [4,5], and an environment generally less favorable for many disease vectors [7,8], but can also modify land uses to expose humans to new pathogens and vectors [57]. While global and regional-scale results here show a general trend of decreased *P*. *vivax* transmission in urban areas, the heterogeneous impacts of urbanization on *P*. *vivax* malaria transmission at the city scale found in these analyses support increasing concerns of urban malaria problems in developing countries. Urbanization in these regions is often associated with poverty, poor water supplies and sanitation in peri-urban areas, providing breading sites for certain vectors [12]. Although malaria vectors are generally not favoured by urban environments, there is evidence highlighting the potential of malaria vectors in adapting to urban environments [58-60]. For example, *Anopheles gambiae s*.*s*. was found breeding in polluted water bodies in Lagos, Nigeria [59]. Furthermore, many studies suggested that urban agriculture is another important source for providing favourable breeding sites for malaria vectors in cities [61-64]. Increased malaria prevalence is often found in communities within a distance of 1 km from irrigated urban agriculture in Accra, Ghana [64], for example. Thus, malaria transmission in urban areas exhibits considerable spatial heterogeneity both between and within cities, depending on factors such as proximity to possible vector breeding habitats, urbanization level and socio-economic status [7,65]. Future work should aim to elucidate these drivers through examination of the disparity of *P*. *vivax* malaria transmission between and within cities using detailed household prevalence surveys and higher resolution urban maps.

In general, the results here highlight a consistent relationship at large scales between urban areas and lower *P*. *vivax* transmission, mirroring results found for *P*. *falciparum*, and pointing towards global declines in *P*. *vivax* transmission as urbanization permanently alters the receptivity of many areas. The findings suggest that these trends will likely continue to catalyze malaria declines on the path to a malaria free future.

## Abbreviations

Africa+: Africa, Saudi Arabia and Yemen; Asia+: Central and South East Asia; DVS: Dominant vector species; GRUMP: Global Rural Urban Mapping Project; GRUMP-UE: GRUMP urban extent; MAP: Malaria Atlas Project; *Pf*PR: *P*. *falciparum* parasite rate; *Pv*API: *P*. *vivax* annual parasite incidence; *Pv*PR: *P*. *vivax* parasite rate; *Pv*MECs: *P*. *vivax* malaria endemic countries.

## Competing interests

The authors declare that they have no competing interests.

## Authors' contributions

AJT conceived the analyses. QQ and AJT developed the study design and QQ conducted the analyses. CAG, CMM and IRF gathered and processed the malaria prevalence data. PWG, CAG and SIH undertook construction of the vivax limits and dominant vector species dataset. All authors contributed to the writing of the manuscript. All authors read and approved the final manuscript.

## Supplementary Material

Additional file 1 **Results of Wilcoxon Signed Rank tests on *Pv *PR values between GRUMP-UE defined urban (U) and rural(R) survey pairs for the dominant *Anopheles *vectors of human malaria.** Click here for file

Additional file 2 **Results of Wilcoxon Signed Rank tests on *Pv *PR values between MODIS defined urban (U) and rural(R) survey pairs for continents, countries and the World.** Click here for file
