# Supplementary material for: The effects of urbanization on global Plasmodium vivax malaria transmission
Source: Malar J. 2012 Dec 5;11:403. doi: 10.1186/1475-2875-11-403 (PMC3528462; doi:10.1186/1475-2875-11-403)
Supplement: Additional file 1 — Results of Wilcoxon Signed Rank tests on Pv PR values between GRUMP-UE defined urban (U) and rural(R) survey pairs for the dominant Anopheles vectors of human malaria. [file 1475-2875-11-403-S1.docx]

**Additional file 1:**

**Table A1.1: Results of Wilcoxon Signed Rank tests on *Pv*PR values between GRUMP-UE defined urban (U) and rural(R) survey pairs for the dominant *Anopheles* vectors of human malaria in Asia-Pacific region**

| **Dominant *Anopheles* vector species** | **No. pairs** | **U>R** | | **U<R** | | **U=R** | **Z** | **P-value** |
| --- | --- | --- | --- | --- | --- | --- | --- | --- |
|  |  | **No. pairs** | **Rank sum** | **No. pairs** | **Rank sum** | **No. pairs** |  |  |
| *An. aconitus* | 467 | 56 | 15006 | 351 | 68022 | 60 | -11.163 | <0.001*** |
| *An. annularis* | 453 | 57 | 15184 | 342 | 64616 | 54 | -10.722 | <0.001*** |
| *An. balabacensis* | 18 | 4 | 25 | 6 | 30 | 8 | -0.255 | 0.838 |
| *An. barbirostris*^#^ | 462 | 53 | 14200 | 348 | 66401 | 61 | -11.239 | <0.001*** |
| *An. culicifacies*^#^ | 129 | 18 | 1467 | 111 | 6918 | 0 | -6.407 | <0.001*** |
| *An. dirus*^#^ | 95 | 7 | 428 | 87 | 4037 | 1 | -6.805 | <0.001*** |
| *An. farauti*^#^ | 104 | 28 | 1370 | 76 | 4090 | 0 | -4.410 | <0.001*** |
| *An. flavirostris* | 317 | 41 | 7583 | 217 | 25878 | 59 | -7.645 | <0.001*** |
| *An. fluviatilis*^#^ | 51 | 10 | 315 | 41 | 1011 | 0 | -3.262 | 0.001*** |
| *An. koliensis* | 86 | 25 | 1003 | 61 | 2738 | 0 | -3.735 | <0.001*** |
| *An. lesteri* | 24 | 8 | 127 | 12 | 173 | 4 | -0.657 | 0.527 |
| *An. leucosphyrus & An. latens* | 149 | 26 | 2584 | 114 | 7286 | 9 | -4.890 | <0.001*** |
| *An. maculatus group* | 504 | 65 | 19156 | 378 | 79190 | 61 | -11.133 | <0.001*** |
| *An. minimus*^#^ | 91 | 8 | 480 | 82 | 3615 | 1 | -6.307 | <0.001*** |
| *An. punctulatus*^#^ | 104 | 28 | 1363 | 76 | 4097 | 0 | -4.433 | <0.001*** |
| *An. sinensis*^#^ | 318 | 56 | 11525 | 256 | 37303 | 6 | -8.082 | <0.001*** |
| *An. stephensi* | 93 | 20 | 1106 | 69 | 2899 | 4 | -3.668 | <0.001*** |
| *An. subpictus*^#^ | 233 | 43 | 5692 | 179 | 19061 | 11 | -6.977 | <0.001*** |
| *An. sundaicus*^#^ | 27 | 4 | 47 | 17 | 184 | 6 | -2.381 | 0.018** |

(^#^) denotes that a vector species is now recognized as a species complex. (*) denotes the significant level of the test (***=P<0.01, **=P<0.05, *=P<0.1).

**Table A1.2: Results of Wilcoxon Signed Rank tests on *Pv*PR values between GRUMP-UE defined urban (U) and rural(R) survey pairs for the dominant *Anopheles* vectors of human malaria in Africa, Europe and the Middle East**

| **Dominant *Anopheles* vector species** | **No. pairs** | **U>R** | | **U<R** | | **U=R** | **Z** | **P-value** |
| --- | --- | --- | --- | --- | --- | --- | --- | --- |
|  |  | **No. pairs** | **Rank sum** | **No. pairs** | **Rank sum** | **No. pairs** |  |  |
| *An. arabiensis* | 422 | 32 | 1360 | 84 | 5426 | 306 | -5.601 | <0.001*** |
| *An. funestus* | 207 | 20 | 771 | 73 | 3600 | 114 | -5.420 | <0.001*** |
| *An. gambiae* | 115 | 1 | 2 | 3 | 8 | 111 | -1.095 | 0.361 |
| *An. melas* | 4 | 0 | 0 | 2 | 3 | 2 | -1.342 | 0.371 |
| *An. moucheti* | 20 | 0 | 0 | 2 | 3 | 18 | -1.342 | 0.371 |
| *An. nili*^#^ | 227 | 11 | 130 | 20 | 366 | 196 | -2.312 | 0.021** |
| *An. sacharovi* | 4 | 0 | 0 | 0 | 0 | 4 | NA | NA |
| *An. sergentii* | 34 | 6 | 31 | 13 | 159 | 15 | -2.576 | 0.011** |
| *An. superpictus* | 65 | 22 | 927 | 35 | 726 | 8 | 0.798 | 0.427 |

(^#^) denotes that a vector species is now recognized as a species complex. (*) denotes the significant level of the test (***=P<0.01, **=P<0.05, *=P<0.1).

**Table A1.3: Results of Wilcoxon Signed Rank tests on *Pv*PR values between GRUMP-UE defined urban (U) and rural(R) survey pairs for the dominant *Anopheles* vectors of human malaria in the Americas**

| **Dominant *Anopheles* vector species** | **No. pairs** | | **U>R** | | **U<R** | | **U=R** | **Z** | **P-value** |
| --- | --- | --- | --- | --- | --- | --- | --- | --- | --- |
|  |  |  | **No. pairs** | **Rank sum** | **No. pairs** | **Rank sum** | **No. pairs** |  |  |
| *An. albimanus* | | 21 | 5 | 74 | 13 | 97 | 3 | -0.501 | 0.628 |
| *An. albitarsis* | | 28 | 17 | 235 | 7 | 65 | 4 | 2.429 | 0.016** |
| *An. aquasalis* | | 2 | 1 | 2 | 1 | 1 | 0 | 0.447 | 1.000 |
| *An. darlingi* | | 28 | 19 | 229 | 5 | 71 | 4 | 2.257 | 0.025** |
| *An. marajoara* | | 24 | 16 | 168 | 4 | 42 | 4 | 2.352 | 0.020** |
| *An. nuneztovari* | | 23 | 16 | 153 | 3 | 37 | 4 | 2.334 | 0.021** |
| *An. pseudopunctipennis* | | 22 | 6 | 95 | 13 | 95 | 3 | 0 | 1.000 |

(*) denotes the significant level of the test (***=P<0.01, **=P<0.05, *=P<0.1).
