# Supplementary material for: The effects of urbanization on global Plasmodium vivax malaria transmission
Source: Malar J. 2012 Dec 5;11:403. doi: 10.1186/1475-2875-11-403 (PMC3528462; doi:10.1186/1475-2875-11-403)
Supplement: Additional file 2 — Results of Wilcoxon Signed Rank tests on Pv PR values between MODIS defined urban (U) and rural(R) survey pairs for continents, countries and the World. [file 1475-2875-11-403-S2.docx]

**Additional file 2**

**Table A2.1: Results of Wilcoxon Signed Rank tests on *Pv*PR values between MODIS defined urban (U) and rural(R) survey pairs for continents, countries and the World**

| **Region** | **No. pairs** | **U>R** | | **U<R** | | **U=R** | **Z** | **P-value** |
| --- | --- | --- | --- | --- | --- | --- | --- | --- |
|  |  | **No. pairs** | **Rank sum** | **No. pairs** | **Rank sum** | **No. pairs** |  |  |
| Africa+ | 192 | 11 | 75 | 3 | 30 | 178 | 1.412 | 0.167 |
| *Ethiopia* | *7* | *5* | *17* | *1* | *4* | *1* | *1.363* | *0.208* |
| *Sudan* | *146* | *6* | *22* | *1* | *6* | *139* | *1.352* | *0.205* |
| *Zambia* | *35* | *0* | *0* | *0* | *0* | *35* | *NA* | *NA* |
| Americas | 15 | 8 | 60 | 7 | 60 | 0 | 0 | 1.000 |
| *Brazil* | *8* | *5* | *15* | *3* | *21* | *0* | *-0.420* | *0.742* |
| Asia+ | 138 | 20 | 1604 | 95 | 5066 | 23 | -4.831 | <0.001*** |
| *Afghanistan* | *19* | *6* | *76* | *12* | *95* | *1* | *-0.413* | *0.695* |
| *Indonesia* | *94* | *8* | *500* | *72* | *2740* | *14* | *-5.372* | *<0.001**** |
| *India* | *8* | *2* | *3* | *0* | *NA* | *6* | *1.342* | *0.371* |
| *Vietnam* | *8* | *3* | *14* | *4* | *14* | *1* | *0* | *1.000* |
| World | 345 | 39 | 3378 | 105 | 7062 | 201 | -3.674 | <0.001*** |
